# Impact of Thoracic Injury on Traumatic Brain Injury Outcome

**DOI:** 10.1371/journal.pone.0074204

**Published:** 2013-09-03

**Authors:** Dawei Dai, Qiang Yuan, Yinfeng Sun, Fang Yuan, Zuopeng Su, Jun Ding, Hengli Tian

**Affiliations:** 1 Department of Neurosurgery, Shanghai 6th People’s Hospital, Shanghai Jiao Tong University, Shanghai, China; 2 Department of Hyperbaric Oxygen, Shanghai 6th People’s Hospital, Shanghai Jiao Tong University, Shanghai, China; Northwestern University Feinberg School of Medicine, United States of America

## Abstract

**Background:**

To assessed the significance of thoracic injury on the 30-day mortality and outcome of traumatic brain injury (TBI).

**Methods:**

TBI patients admitted to our department were retrospectively evaluated. We developed two prognostic models based on admission predictors with logistic regression analysis to assess the significance of thoracic injuries in determining the 30-day mortality and outcome. The internal validity of the models was evaluated with the bootstrap re-sampling technique. We also validated the models in an external series of 165 patients that collected from our center. Discriminative ability was evaluated with C statistic. Calibrative ability was assessed with the Hosmer-Lemeshow test (H-L test).

**Results:**

Among 505 TBI patients admitted, 102 (20.2%) had thoracic injuries. Patients with a PCS ≥6 had a 3.142 and 8.065 times higher odds of mortality and poor outcome compared with patients with a PCS <6, respectively. Any one-score increase of the TTS had a 1.193 times higher odds of a poor outcome (p = 0.017). The predictive model for mortality and 30-day functional outcome both had good accuracy (AUC: 0.875; 95% confidence interval [CI], 0.841–0.910 and AUC: 0.888; 95%CI, 0.860–0.916, respectively). Internal validation showed no over optimism in any of the two models’ predictive C statistics (C statistic 0.872 for 30-day mortality and C statistic 0.884 for the 30-day neurological outcome). The external validation confirmed the discriminatory ability of these models (C statistic 0.949 (95%CI: 0.919–0.980) for 30-day mortality and C statistic 0.915 (95%CI: 0.868–0.963) for the 30-day neurological outcome). The calibration was also good for patients from the validation population (H-L test p>0.05).

**Conclusion:**

Thoracic injury diagnosed by CT has a negative impact on the 30-day mortality and functional outcome of TBI patients. The extent of PC and the TTS are the predictors for TBI outcome.

## Introduction

Traumatic brain injury (TBI) is the leading cause of morbidity and disability among trauma groups, and is responsible for a notable proportion of all traumatic deaths, particularly in young adults [1,2]. Additionally, TBI patients have a high proportion of associated thoracic injuries. Pulmonary contusion (PC) is involved in 29% of TBI patients [3]. Despite advances in pulmonary care and ventilator management, TBI has been described as contributing to higher mortality and morbidity in patients with thoracic injuries, particularly in children [4,5].

Chest computed tomography (CT) is systematically performed in TBI patients at many trauma centers [6]. Performing chest CT allows for an easy diagnosis of thoracic injuries, which can generate worsening of gas exchanges and acute respiratory failure. This can further result in hypoxemia, which may aggravate TBI despite adequate management of patients. Several studies have demonstrated that hypoxemia constitutes a secondary insult that could be related to poor outcome in TBI patients [7–9]. The association between TBI and thoracic injuries has been described as a vicious circle, leading to the constitution of secondary insult. Therefore, knowledge of outcome determinants is important because it may provide early accurate information to the patients’ next of kin and allow better allocation of Neonatal Intensive Care Unit (NICU) resources. However, some data from analyses of outcome determinants in TBI patients have not been in agreement with previous assertions. Moreover, previous studies did not further distinguish the impact of different types of chest injuries on TBI prognosis. Therefore, the objective of the present study was to determine the impact of thoracic injuries, as diagnosed by CT, on the morbidity and mortality of patients with TBI.

## Patients and Methods

### Data source

All TBI patients admitted to the Sixth People’s Hospital affiliated with Shanghai Jiao Tong University from January 2010 through May 2012 were retrospectively evaluated. This study was approved by the Scientific and Ethical Committee of Shanghai JiaoTong University. This study was approved by the Scientific and Ethical Committee of Shanghai JiaoTong University. All participants provide their written informed consent to participate in this study, and the consent procedure was approved by the Scientific and Ethical Committee of Shanghai JiaoTong University. The next of kin, carer takers or guardians consented on the behalf of participants whose capacity to consent was compromised. TBI was confirmed by CT. Exclusion criteria included a body region other than the brain and chest with an Abbreviated Injury Severity score (AIS) ≥3 (exclusion of other significant systemic injuries) and patients with spinal cord injuries. Additionally, all patients included in the present study underwent chest CT during their initial assessment at the emergency department and the chest CT and thoracic injuries were followed up. Thoracic injury was defined as the presence of PC or laceration, and/or fractured ribs, and/or pneumothorax or hemothorax.

Data extracted from patients’ medical records included age, sex, cause of injury, head and chest AIS scores, Injury Severity Score (ISS), Glasgow Coma Scale (GCS) score at admission, types of thoracic injuries, pupillary size, and light response. Patients were followed for 30 days after injury or until death if the latter occurred before the end of the 30-day period. The 30-day neurological outcome was determined according to the Glasgow Outcome Score (GOS) [10]. Under this rating system, a GOS of 1 indicated death; a GOS of 2 indicated a persistent vegetative state; a GOS of 3 indicated severe disability (conscious but disabled); a GOS of 4 indicated moderate disability (disabled but independent); and a GOS of 5 indicated excellent recovery with a return to baseline functional status. Prognostic evaluations were determined using the GOS assessment 6 months after the trauma. GOS evaluations were performed by physicians either in person or via telephone. A GOS of 1–3 was categorized as an "unfavorable" outcome, while a score of 4–5 was deemed a "favorable" outcome.

All cases were evaluated for the presence or absence of contusion, atelectasis, pulmonary laceration, pneumothorax, hemothorax, and fractured ribs. PC on CT was defined as an area of increased lung opacity without bronchovascular crowding. The extent of PC was measured using the Pulmonary Contusion Score (PCS) that was developed by Tyburski [4]. This score divided each lung field into upper, middle, and lower thirds. For the PCS, each third received a score of 1–3 based on the density of the affected lung. In this manner, if a contusion encompassed a small area of the lower third, it would receive a PCS of 1. Complete opacification of the lower third of one lung would receive a PCS of 3, and opacification of the entire lung would receive a PCS of 9. Thus, a single CT could receive a PCS between 1 and 18. Patients with PC were compared to those without PC.

Patients with thoracic injuries were also assessed using the Thoracic Trauma Severity score (TTS; Table 1). The TTS is based on five anatomical and physiologic parameters: PO_2_/FiO_2_, rib fractures, PC, pleural lesion, and age [11]. Each parameter is assigned a value of 0–5 and subsequently summed. The TTS score ranges from 0 to 25. Additionally, the number of fractured ribs and pneumothorax or hemothorax were also recorded.

**Table 1:** Thoracic Trauma Severity score.

Grade	Pao_2_/FIO_2_	Rib Fracture	Contusion	Pleural Involvement	Age (y)	Points
0	>400	0	None	None	<30	0
I	300-400	1-3	1 lobe, unilateral	PT	30-41	1
II	200-300	3-6	1 lobe, bilateral or 2 lobes unilateral	HT/HPT unilateral	42-54	2
III	150-200	>3 bilateral	<2 lobes bilateral	HT/HPT bilateral	55-70	3
IV	<150	Flail chest	≥2 lobes bilateral	TPT	>70	5

PT, pneumothorax; HT, hemothorax; HPT, hemopneumothorax; TPT, tension pneumothorax

For calculation of the total score, all categories are summed up. A minimum value of 0 points and a maximum value of 25 points can be achieved.

For external validation of the models, 165 TBI patients with the same inclusion criteria and similar treatment regimen from June 2012 to April 2013 were collected at our hospital.

**Figure 1:**
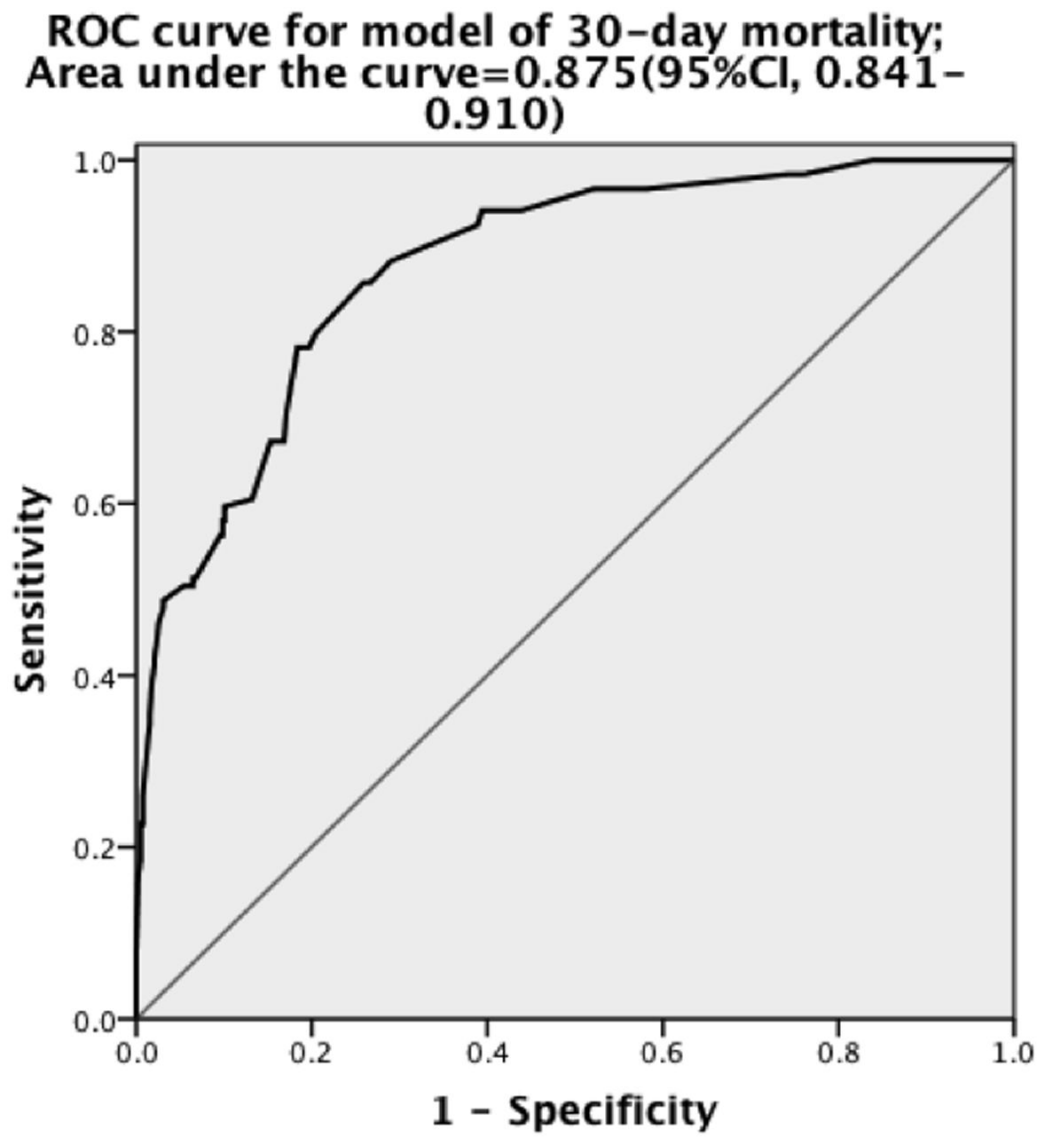
Receiver operating characteristic (ROC) curve for prediction of mortality estimated using multiple logistic regression. Predictive variables for this equation are age, injury severity score ≥25, pulmonary contusion score (PCS) ≥6, GCS (Glasgow Coma Scale) group, and pupillary size and light response with an AUC of 0.875, indicating good accuracy.

**Table 2:** Clinical features of patients classified by death.

	Survived (n = 386)	Died (n = 119)	*p*-value
GCS	10.6 (3.4)	6.3 (3.2)	<0.001
Age in years	46.2 (17.2)	57.2 (18.9)	<0.001
ISS	15.2 (7.0)	22.3 (10.6)	<0.001
Head AIS	3.4 (0.6)	4.1 (0.7)	<0.001
Chest AIS	0.4 (0.9)	0.8 (1.2)	0.002
PCS	0.4 (1.3)	1.2 (2.3)	<0.001
Fractured ribs	49 (12.7%)	17 (14.3%)	0.653
Number of fractured ribs	0.39 (1.22)	0.42 (1.14)	0.787

GCS, Glasgow Coma Scale; ISS, Injury Severity Score; AIS, Abbreviated Injury Scale; PCS, Pulmonary Contusion Score. *p*-value of continuous variables generated by Student’s *t*-test and *p*-value of categorical variables generated by χ^2^ or Fisher’s exact test.

### Statistical Analysis

Student’s *t*-tests were used for continuous variables, χ^2^ or Fisher’s exact tests were used for categorical variables, and logistic regression analysis was used for multivariate analysis. Associations between various parameters extracted from TBI patients’ medical records and 30-day mortality or 30-day functional outcomes were evaluated using univariate analyses followed by multiple logistic regressions. A prediction model was also established using multiple logistic regression. Receiver operating characteristic (ROC) and area under the curve (AUC) analyses were used to determine the adequacy of the prediction models. A *p*-value less than 0.05 was deemed to indicate statistical significance.

The internal validation of the models was assessed with the bootstrap re-sampling technique. Regression models were estimated in 200 bootstrap samples because 200 bootstraps are often suf- ficient to obtain stable estimates. Each re-sampling could be recognized as a replacement for the original samples. For each of the 200 bootstrap samples, we refitted and tested the model on the original sample to obtain an estimate of the predictive accuracy as corrected for bias. Discrimination and calibration were assessed to indicate the performance of the models for the external patients. Calibration was further assessed graphically by plotting observed outcomes against the predicted probabilities. A smooth, nonparametric calibration line created by the Lowess algorithm was used to graphically assess the models’ calibrative abilities [12]. The H-L test used the hl.ext R code written by Steyerberg [13]. In the H-L tests, p values less than 0.05 were considered statistically significant. The statistical package used for analyses was R for Mac Version 2.6.2 (The R Foundation for Statistical Computing).

**Figure 2:**
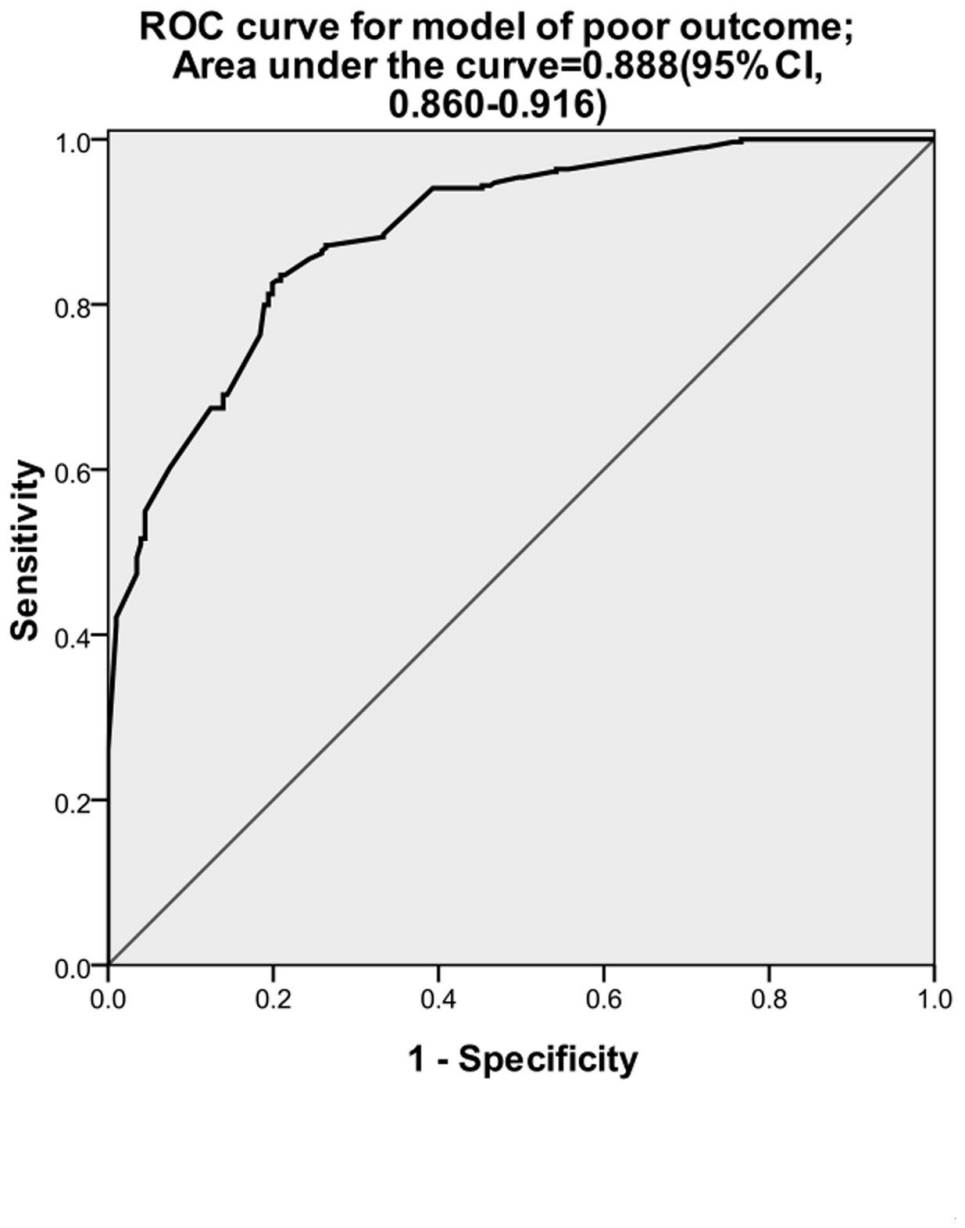
ROC curve for prediction of poor outcome estimated using multiple logistic regression. Predictive variables for this equation are age, head AIS >3, PCS ≥6, GCS group, and TTS score with an AUC of 0.888, indicating good accuracy.

## Results

### Demographics and general characteristics

A total of 505 adults with TBI met the inclusion criteria and were admitted to our general hospital between January 1, 2010 and May 31, 2012. The study patients included 395 men (78.2%) with a mean age of 48.8 ± 18.2 years old and ISSs ranging from 2 to 54 (mean, 17) and GCSs ranging from 3 to 15 (mean, 10). Of these, 102 patients had thoracic injuries, 66 (13.1%) patients had fractured ribs, 68 (13.5%) had PC, 15 (3.0%) had pneumothorax, and 4 had hemothorax (0.8%). TBIs caused by traffic accidents accounted for the leading cause of injury (58.8%). For TBI patients with thoracic injuries, traffic accidents were the leading cause of injury (62.7%). However, TBI patients with thoracic injuries had a higher percentage of falls from a height than patients without thoracic injuries (22.5% vs. 16.9%).

### Predictors of mortality and 30-day outcome

Age, ISS, head and chest AIS scores, and PCS were significantly higher in the group with patients who died during the 30-day period, whereas the GCS score on admission was significantly lower in the group with patients who died (Table 2). Univariate analysis identified that a lower GCS score, older age, abnormal pupillary size and light response on one or two sides, TTS score, ISS ≥25, head AIS >3, PCS ≥6, and presence of pneumothorax or hemothorax were predictors of death (Table 3). Multiple logistic regression models showed that the GCS, age, abnormal pupillary size and light response, ISS ≥25, and PCS ≥6 were predictors of 30-day mortality (Table 4). Any one grade increase in age had a 3.811 times higher odds of 30-day mortality (p < 0.001; for example, a patient aged >65 years had a 3.811 times higher odds of mortality at follow-up as compared with a patient aged between 41 and 54 years). Patients with a PCS ≥6 had a 3.142 times higher odds of mortality at follow-up compared to patients with a PCS <6. Patients with an ISS ≥25 had a 2.564 times higher odds of mortality at follow-up compared to patients with an ISS <25. Any one grade increase in the GCS score at admission and abnormal pupillary size and light response had a 2.513 and 2.112 times higher odds of mortality, respectively. The TTS, a head AIS >3, and the presence of pneumothorax or hemothorax were not significantly associated with mortality (p > 0.05).

**Table 3:** Factors analyzed as predictors of the 30-day mortality.

	Survived (n = 386)	Died (n = 119)	*p*-value
GCS			<0.001
3–5	39 (37.5%)	65 (62.5%)	
6–8	89 (75.4%)	29 (24.6%)	
9–12	80 (81.6%)	18 (18.4%)	
13–15	178 (96.2%)	7 (3.8%)	
Age in years			<0.001
≤40	156 (86.7%)	24 (13.3%)	
41–64	169 (75.4%)	55 (24.6%)	
≥65	61 (60.4%)	40 (39.6%)	
Gender			0.044
Male	294 (74.4%)	101 (25.6%)	
Female	92 (83.6%)	18 (16.4%)	
Cause			0.207
Traffic accidents	233 (78.5%)	64 (21.5%)	
Fall from a height	65 (71.4%)	26 (28.6%)	
Stumble and fall	67 (72.0%)	26 (28.0%)	
Others	21 (87.5%)	3 (12.5%)	
Pupillary size and light response			<0.001
Normal	347 (83.0%)	71 (17.0%)	
Abnormal on one side	33 (78.6%)	9 (21.4%)	
Abnormal on two sides	6 (13.3%)	39 (86.7%)	
Thoracic Trauma Severity Score			0.001
Mean (SD)	1.0 (2.3)	1.9 (3.2)	
ISS			<0.001
<25	331 (83.0%)	68 (17.0%)	
≥25	55 (51.9%)	51 (48.1%)	
Head AIS			<0.001
≤3	223 (89.6%)	26 (10.4%)	
>3	163 (63.7%)	93 (36.3%)	
Chest AIS			0.096
<3	359 (77.4%)	105 (22.6%)	
≥3	27 (65.9%)	14 (34.1%)	
PCS			<0.001
<6	377 (78.5%)	103 (21.5%)	
≥6	9 (36.0%)	16 (64.0%)	
Fractured ribs			0.925
<4	369 (76.4%)	114 (23.6%)	
≥4	17 (77.3%)	5 (22.7%)	
Pneumothorax or hemothorax			0.045
None	378 (77.1%)	112 (22.9%)	
Pneumothorax	5 (45.5%)	6 (54.5%)	
Hemopneumothorax or hemothorax	3 (75.0%)	1 (25.0%)	

GCS, Glasgow coma scale; ISS, Injury Severity Score; AIS, Abbreviated Injury Scale; PCS, Pulmonary Contusion Score. *p*-value of continuous variables generated by Student’s *t*-test and *p*-value of categorical variables generated by χ^2^ or Fisher’s exact test.

**Figure 3:**
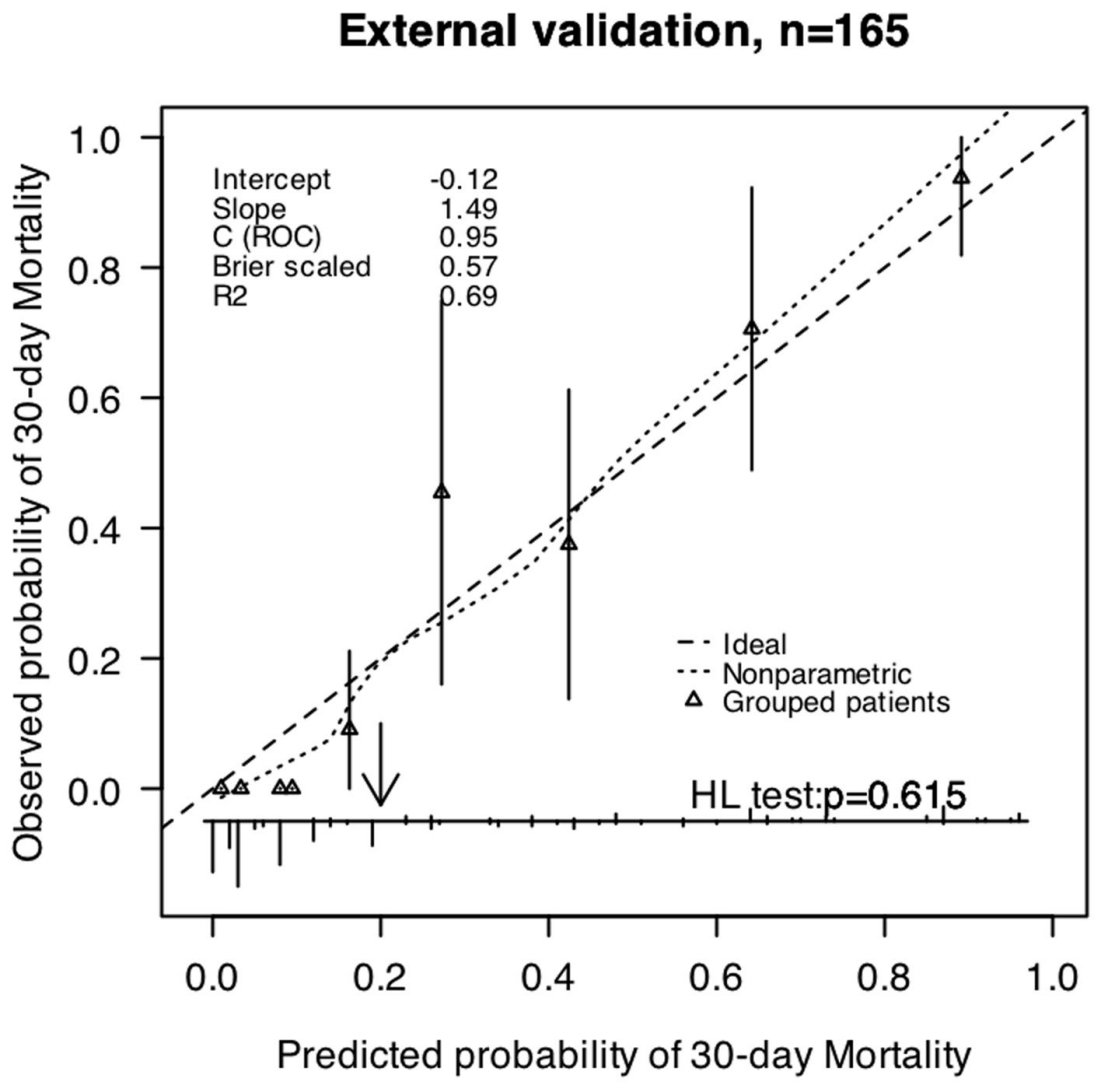
Validation of the prognostic models for 30-day mortality in validation patients (n = 165). The smooth dash curves reflect the relation between observed probability of mortality and predicted probability of mortality. The triangles indicate the observed frequencies by deciles of predicted probability.

**Table 4:** Multiple logistic regression model related to the 30-day mortality of TBI patients.

	*p*	OR (95% CI)
ISS ≥25	0.003	2.564 (1.372–4.792)
PCS ≥6	0.032	3.142 (1.104–8.942)
Age	<0.001	3.811 (2.510–5.788)
GCS	<0.001	0.398 (0.295–0.535)
Pupillary size and light response	0.001	2.112 (1.364–3.270)

GCS, Glasgow Coma Scale; ISS, Injury Severity Score; AIS, Abbreviated

Injury Scale; PCS, Pulmonary Contusion Score; OR, odds ratio; CI, confidence interval.

**Figure 4:**
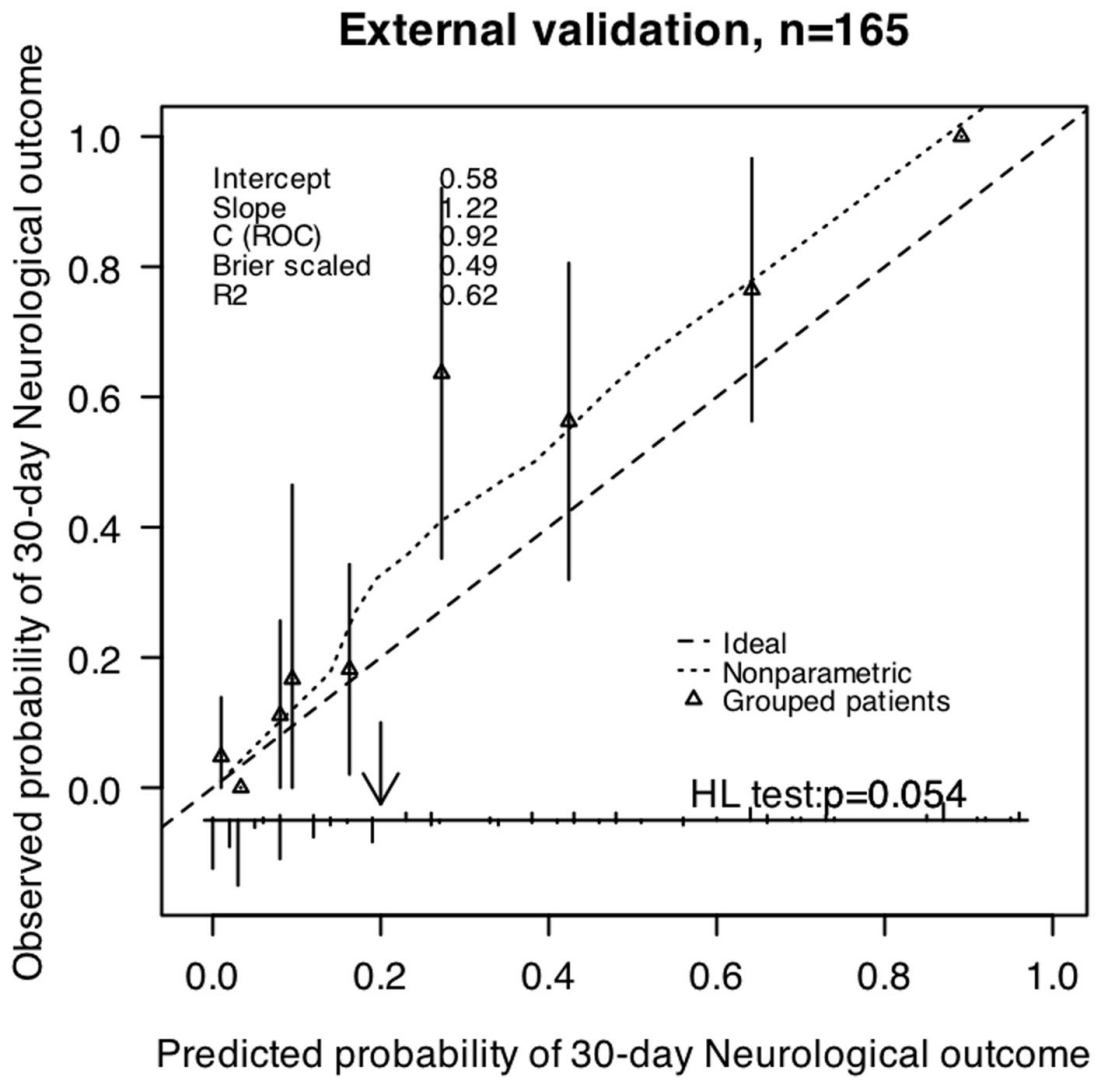
Validation of the prognostic models for 30-day outcome in validation patients (n = 165). The smooth dash curves reflect the relation between observed probability of outcome and predicted probability of outcome. The triangles indicate the observed frequencies by deciles of predicted probability.

**Table 5:** Clinical features of patients classified by functional outcome.

	Poor outcome (n = 201)	Good outcome (n = 304)	*p*-value
GCS	6.8 (3.2)	11.4 (3.0)	<0.001
Age in years	55.5 (18.2)	44.4 (16.9)	<0.001
ISS	20.8 (9.8)	14.3 (6.4)	<0.001
Head AIS	4.0 (0.7)	3.3 (0.6)	<0.001
Chest AIS	0.8 (1.2)	0.3 (0.8)	<0.001
PCS	1.1 (2.3)	0.2 (0.9)	<0.001
Fractured ribs	36 (17.9%)	30 (9.9%)	0.009
Number of fractured ribs	0.55 (1.44)	0.29 (1.00)	0.030

GCS, Glasgow Coma Scale; ISS, Injury Severity Score; AIS, Abbreviated Injury Scale; PCS, Pulmonary Contusion Score. *p*-value of continuous variables generated by Student’s *t*-test and *p*-value of categorical variables generated by χ^2^ or Fisher’s exact test.

**Table 6:** Factors analyzed as predictors of the 30-day functional outcome.

	Poor outcome (n = 201)	Good outcome (n = 304)	*p*-value
GCS			<0.001
3–5	88 (84.6%)	16 (15.4%)	
6–8	60 (50.8%)	58 (49.2%)	
9–12	36 (36.7%)	62 (63.3%)	
13–15	17 (9.2%)	168 (90.8%)	
Age			<0.001
≤40	46 (25.6%)	134 (74.4%)	
41–64	93 (41.5%)	131 (58.5%)	
≥65	62 (61.4%)	39 (38.6%)	
Gender			0.135
Male	164 (41.5%)	231 (58.5%)	
Female	37 (33.6%)	73 (66.4%)	
Cause			0.121
Traffic accidents	189 (63.6%)	108 (36.4%)	
Fall from a height	46 (50.5%)	45 (49.5%)	
Stumble and fall	53 (57.0%)	40 (43.0%)	
Others	16 (66.7%)	8 (33.3%)	
Pupillary size and light response			<0.001
Normal	141 (33.7%)	277 (66.3%)	
Abnormal on one side	18 (42.9%)	24 (57.1%)	
Abnormal on two sides	42 (93.3%)	3 (6.7%)	
Thoracic Trauma Severity Score			<0.001
Mean (SD)	2.0 (3.2)	0.7 (1.9)	
ISS			<0.001
<25	128 (32.1%)	271 (67.9%)	
≥25	73 (68.9%)	33 (31.1%)	
Head AIS			<0.001
≤3	52 (20.9%)	197 (79.1%)	
>3	149 (58.2%)	107 (41.8%)	
Chest AIS			0.004
<3	176 (37.9%)	288 (62.1%)	
≥3	25 (61.0%)	16 (39.0%)	
PCS			<0.001
<6	178 (37.1%)	302 (62.9%)	
≥6	23 (92.0%)	2 (8.0%)	
Fractured ribs			0.149
<4	189 (39.1%)	294 (60.9%)	
≥4	12 (54.5%)	10 (45.5%)	
Pneumothorax or hemothorax			
None	190 (38.8%)	300 (61.2%)	0.020
pneumothorax	8 (72.7%)	3 (27.3%)	
Hemopneumothorax or hemothorax	3 (75.0%)	1 (25.0%)	

GCS, Glasgow Coma Scale; ISS, Injury Severity Score; AIS, Abbreviated Injury Scale; PCS, Pulmonary Contusion Score. *p*-value of continuous variables generated by Student’s *t*-test and *p*-value of categorical variables generated by χ^2^ or Fisher’s exact test.

Age, ISS, head and chest AIS, PCS, and the number of fractured ribs were significantly higher in the group with poor outcome whereas the GCS score on admission was statistically significantly lower in the group with poor outcome (Table 5). Univariate analysis identified that a lower GCS score, older age, abnormal pupillary size and light response on one or two sides, TTS score, ISS ≥25, head AIS >3, PCS ≥6, and presence of pneumothorax or hemothorax were also predictors of 30-day poor outcome (Table 6). Multiple logistic regression models showed that the GCS, age, TTS score, head AIS, and PCS ≥6 were predictors of poor outcome (Table 7). Any one grade increase of age had a 3.759 times higher odds of a poor outcome (*p* = 0.017). For example, a patient aged >65 years had a 3.759 times higher odds of a poor outcome at follow-up as compared with a patient aged between 41 and 54 years. Patients with a PCS ≥6 had an 8.065 times higher odds of a poor outcome at follow-up compared to a patient with PC<6. Any one score increase of the Thoracic Trauma Severity score has 1.193 times higher odds of a poor outcome (*p* = 0.017). Patients with a head AIS >3 had a 1.980 times higher odds of a poor outcome at follow-up compared to patients with a head AIS ≤3. Any one grade increase in the GCS score at admission had a 3.575 times higher odds of a poor outcome. An ISS ≥25 or the presence of pneumothorax or hemothorax was not significantly associated with a poor outcome (*p* > 0.05).

**Table 7:** Multiple logistic regression model related to the 30-day poor outcome of TBI patients.

	*p*	OR (95% CI)
Thoracic Trauma Severity Score	0.002	0.838 (0.750–0.936)
PCS ≥6	0.027	0.124 (0.019–0.790)
Age	<0.001	0.266 (0.182–0.387)
Head AIS >3	0.019	0.505 (0.286–0.893)
GCS	<0.001	3.575 (2.701–4.732)

GCS, Glasgow coma scale; ISS, Injury Severity Score; AIS, Abbreviated

Injury Scale; PCS, Pulmonary Contusion Score; OR, odds ratio; CI, confidence interval.

### Predictive models performance and validation

Multivariate logistic regression was conducted to analyze the most accurate predictive model for mortality. Figure 1 shows the ROC curve for the resulting equation, which is based on age, GCS, PCS, abnormal pupillary size and light response, and ISS. By integrating these variables, the predictive model had an AUC of 0.875 (95% CI, 0.841–0.910), indicating good accuracy. Multivariate logistic regression was also conducted to analyze the most accurate predictive model for poor outcome. Figure 2 shows the ROC curve for the resulting equation, which is based on age, GCS, PCS, TTS score, and head AIS. By integrating these variables, the predictive model demonstrated an AUC of 0.888 (95% CI, 0.860–0.916), also indicating good accuracy.

Internal validation showed no over optimism in any of the two models’ predictive C statistics (C statistic 0.872 for 30-day mortality and C statistic 0.884 for the 30-day neurological outcome). The external validation confirmed the discriminatory ability of these models (C statistic 0.949 (95%CI: 0.919–0.980) for 30-day mortality and C statistic 0.915 (95%CI: 0.868–0.963) for the 30-day neurological outcome). The calibration was also good for patients from the validation population (H-L test p>0.05). Therefore, the two models were generalizable and could be used for predicting the outcomes of new patients. Calibration curves for the outcomes are shown in Figure 3, Figure 4.

## Discussion

The aim of the present study was to assess the impact of different types of thoracic injuries on TBI outcome. In our study, we found that thoracic injury is an independent risk factor of 30-day mortality and poor outcome. The ROC curve for the multivariate logistic regression model in which thoracic injuries were summed also demonstrated good accuracy for both mortality and poor outcome.

Chest radiography (CXR) has traditionally been used as a screening method in blunt thoracic trauma. However, a normal CXR upon admission does not exclude thoracic injuries or other significant injuries [14]. Chest CT is more sensitive than conventional CXR in detecting blunt thoracic injuries. Multidetector chest CT offers a more accurate initial evaluation of thoracic injury and provides information concerning pulmonary function and outcome. In our study, all included patients underwent chest CT during their initial assessment at the emergency department and the chest CT and thoracic injuries were followed up.

The present study found that thoracic injury was frequent in patients with TBI and was closely related to TBI outcome. According to our study, the factor most closely related to TBI outcome was the extent of PC. The multiple logistic regression models in our study showed that patients with a PCS ≥6 had a 3.142 times higher odds of mortality at follow-up compared to patients with a PCS <6. Several studies have accessed the influence of PC in head injury. A study performed in children with head injuries showed a worsening of outcome in the presence of PC [5]. The extent of PC within the first 24 h has been correlated with death and morbidity.4 However, a previous study found that the extent of contusion assessed on hospital admission chest roentgenograms was not predictive of mortality [15]. A case-control study performed by Leone et al. found that the overall mortality rate was 41% in severe TBI patients with PC and 34% in severe TBI patients without PC; however, this difference was not statistically significant and the authors ascribed it to the small sample size [3].

The TTS score is based on five anatomical and physiologic parameters: PO_2_/FiO_2_, rib fractures, PC, pleural lesion, and age [11]. Each parameter is assigned a value of 0–5 and subsequently summed. The TTS score ranges from 0 to 25. With increasing score values, more severe thoracic trauma could be assumed; however, the authors of the TTS score have not recommended a specific cut-off value. In our study, the continuous TTS score was included in the regression model and the TTS score was not significantly associated with mortality (*p* > 0.05); however, any one score increase of the TTS score has a 1.193 times higher odds of a poor outcome. Additionally, the presence of pneumothorax or hemothorax was not significantly associated with mortality and poor outcome (*p* > 0.05), a finding that may due to the low incidence rate of pneumothorax or hemothorax (only 3.8%).

Many previous studies that lack more objective measures of PC or thoracic injury have limited ability to predict outcome in variable cohorts of patients. Some have suggested complex mathematical models for quantifying PC or new thoracic trauma scores [16]. Therefore, we believe that using the PCS to quantify PC and using the TTS to quantify the extent of thoracic injury in our study are more objective to assess different types of thoracic injury.

The concept of systemic secondary insults may be the best explanation of thoracic injury related to poor outcome in TBI patients [17–20]. This concept asserts that chest trauma causes respiratory failure with hypoxemia and hypercapnia, aggravating the consequences of head trauma. Many studies have confirmed that chest trauma is related to respiratory dysfunction. Measurement of contusion volume has been described as a determinant of patients with a high risk of complications, particularly acute respiratory distress syndrome (ARDS) [21]. Leone et al. also found that PC had a negative impact on gas exchange.3 PC has been shown to be an independent predictor of required ventilation support and the development of serious complications, such as ARDS and pneumonia [22,23]. One study correlating the volume of PC with the risk of developing ARDS showed that the risk of ARDS is 82% for a patient whose lungs have more than 20% contusion volume versus 22% for a patient whose lungs have less than 20% contusion volume [21].

Other potential confounders were included as predictors in the multivariable models based on clinical importance to TBI outcome: age, GCS score at initial emergency department presentation, abnormal pupillary size and light response, and ISS. Consistent with previous studies, these variables were all related to the TBI outcome. In our study, the significant factors for 30-day mortality and poor outcome are not the same, this difference is consistent with other reports [24].

External validation of prognostic models is essential to assess their generalizability, which indicates how a model performs for new patients. Models often perform worse during external validation than during the initial assessment. In this study, prognostic models for 30-day mortality and 30-day outcome have a C statistic from 0.919 to 0.980 and from 0.868 to 0.963 respectively for validation data and a p value > 0.05, indicating that the models discriminate and calibrate well for validation data. In this study, the models’ high discrimination and calibration in the validation patients demonstrate that the models were stable.

Several limitations exist in our study. First, the study was retrospective in nature as mentioned above. A further well-designed prospective randomized clinical trial is necessary for a more precise result. Second, chest trauma resulting in hypoxemia, constituting a secondary insult, may be the leading cause that aggravates head trauma; however, our study did not further study the relationship between thoracic injury and hypoxemia. Third, significant thoracic injury likely contributes similarly to morbidity and mortality even in patients who did not suffer a TBI. Our study only enrolled patients who suffered TBI combined with thoracic injury, not included patients suffered thoracic injury only. The mortality and morbidity rates different between patients who suffer TBI with thoracic injury and thoracic injury alone should be discussed in future study.

## Conclusion

In conclusion, the present study assessed, for the first time, the effects of different types of thoracic injuries on outcomes in TBI patients. Thoracic injury diagnosed on CT has a negative impact on 30-day mortality and functional outcome of TBI patients. The extent of PC and TTS scores are the predictors for TBI outcome.
